# A PUF-Based Key Storage Scheme Using Fuzzy Vault †

**DOI:** 10.3390/s23073476

**Published:** 2023-03-26

**Authors:** Jinrong Yang, Shuai Chen, Yuan Cao

**Affiliations:** 1 School of Computer Science and Artificial Intelligence, Wuhan University of Technology, Wuhan 430070, China; 2 Rock-Solid Security Lab., Binary Semiconductor Co., Ltd., Suzhou 215000, China; 3 Collage of Internet of Things Engineering, Hohai University, Changzhou 213022, China

**Keywords:** Physical Unclonable Functions, error-tolerant, fuzzy pattern

## Abstract

Physical Unclonable Functions (PUFs) are considered attractive low-cost security anchors in the key generation scheme. The helper data algorithm is usually used to transform the fuzzy responses extracted from PUF into a reproducible key. The generated key can be used to encrypt secret data in traditional security schemes. In contrast, this work shows that the fuzzy responses of both weak and strong PUFs can be used to secretly store the important data (e.g., the distributed keys) directly by an error-tolerant algorithm, Fuzzy Vault, without the traditional encryption algorithm and helper data scheme. The locking and unlocking methods of our proposal are designed to leverage the feature of weak and strong PUFs relatively. For the strong PUFs, our proposal is a new train of thought about how to leverage the advantage of strong PUFs (exponential number of challenge–response pairs) when used in the field. The evaluation was performed on existing weak PUF and strong PUF designs. The unlocking rate and runtime are tested under different parameters and environments. The test results demonstrate that our proposal can reach a 100% unlocking rate by parameter adjustment with less than 1 second of locking time and a few seconds of unlocking time. Finally, the tradeoff between security, reliability, and overhead of the new proposal is discussed.

## 1. Introduction

The Internet of Things (IoT) is pervasively distributed in our daily life. Despite the undeniable benefits, it raises serious security and privacy concerns. The security of IoT has drawn increasing public attention. In IoT devices, the security of traditional secret systems relies on the secret key which is permanently stored in Non-Volatile Memory (NVM). However, storing the secret key safely in NVM is challenging. Firstly, for some resource-constrained IoT devices, it is not affordable to achieve the desired security level that can countermeasure the related attacks, e.g., invasive and semi-invasive attacks. Secondly, the secret keys need to be generated and “burned” into the devices during the production process, which may lead to the so-called supply chain security issue [1]. Therefore, a secure key generation solution that is deployable for resource-constrained IoT devices is required.

Physical Unclonable Functions (PUFs) leverage the unique physical feature of the integrated circuits to do key generation and authentication which can meet the requirements aforementioned [2]. The unique features of PUF are dependent on the nano-scale variations of devices introduced during the manufacturing process, which is random and difficult to be replicated. Based on the number of possible challenges, PUFs can be mainly divided into two classes: weak PUFs and strong PUFs [3]. Weak PUFs have very few challenges or even only one challenge in one instance. When used in the field, the challenge and response pairs (CRPs) or CRP should be kept in secret. Weak PUFs are considered as attractive low-cost security anchors to mitigate some potential pitfalls in NVM-based key storage schemes. On the contrary, strong PUFs usually have an exponential number of CRPs. The exponential CRPs can be used in the CRPs-based identification and authentication scheme [4].

As a promising cryptographic primitive in key generation, authentication, and identification scheme, it is extremely important that the PUFs function correctly during the lifetime of the devices. However, most PUFs exhibit an unreliability problem due to aging and inherent sensitivity to environmental changes. As a remedy to the reliability issue, helper data algorithms [5] are used in practice, which can transform the unique feature of PUFs into a reproducible high-entropy key. In the helper data algorithm, the helper data are generated and stored in NVM during the enrollment phase. The generated helper data are used then to correct the fuzzy response extracted from fuzzy PUFs. The privacy algorithm (e.g., hash algorithm) is used to maximize the entropy of the key. Ref. [5] mentioned that most work on helper data algorithms considers the “reproducibility” and “uniformly distributed” only and ignored the key requirements of “PUFs independency”, “mathematical restrictions” and “full control”.

In the key generation scheme, the key is extracted from PUFs with the help of a helper data algorithm. This hardware-based unique key can then be used to encrypt the important data, e.g., the application key [6,7] by some classical encryption algorithm (e.g., AES and DES). In this study, we show that fuzzy PUFs can be used to protect the short secret data (e.g., the secret key) directly without the classical encryption algorithm. Compared with the key generation scheme based on a helper data algorithm, our scheme can be considered.

Juel and Sudan’s Fuzzy Vault [8] is an error-tolerant cryptographic construction. It is an effective way for biometric template protection in biometric identification [9]. In the Fuzzy Vault algorithm, the fuzzy biometric data can be hidden with secret data in randomly selected chaff points to ensure the biometric template security. Although the biometric data suffer from the noise effects, the error-tolerant feature of Fuzzy Vault allows the identification success. A typical application of Fuzzy Vault is the fingerprint-based Fuzzy Vault [10].

Naturally, the error-tolerant feature of Fuzzy Vault is suitable for fuzzy PUFs. Each PUF instance has unique security features such as fingerprints. Therefore, contrary to protecting the fingerprint templates by the secret data, the security PUF template can be used to protect the secret data in the vault with a higher security level.

This study demonstrates that the short secret data can be locked in a vault leveraging both the fuzzy weak PUFs and fuzzy strong PUFs. Some preprocessing for PUF responses is designed to make PUFs more suitable for the fuzzy vault. The secret data can be unlocked when part of the PUF features remains stable. Our proposal is suitable for the scenario in that the secret keys are generated or shared inside or outside the devices which need to be protected in memory. For example, every time a Digital Certificate is issued (e.g., in HTTPS web site), a private key is generated. The private key needs to be stored in secret or else the security system will be compromised [11,12]. In the Android system, an APP can generate or receive several private/public key pairs and store them in the Android Keystore [13]. The classical methods aforementioned are mainly based on the existing encryption algorithm to secret the private keys. In our proposal, the private keys can be encrypted by the fuzzy hardware “fingerprints”. For weak PUFs, each PUF instance can only be used once to protect one secrete data, because the vault is vulnerable to the correlation attack [14]. On the contrary, for strong PUFs, the exponential number of CRPs can help one PUF instance protect the exponential number of secret data in vaults. With some basic security assumptions, the strong PUFs can avoid the correlation attack and modeling attack in our proposal.

Part of our submitted paper is the extended work and further research of our previous conference paper that was published in AsianHOST [15]. Our key contributions are as follows:

- We demonstrate that the fuzzy PUFs can be used to secretly store important data without the error-correction algorithm, which is a new train of thought about how to use PUFs in an embedded system.
- We implemented the PUF-based Fuzzy Vault in Python. The pre-processing processes for both weak and strong PUFs are designed to make our design more suitable for PUFs.
- The runtime and successful rate of the new proposal are evaluated with one existing weak PUF and one existing strong PUF implementation.
- We discussed the security and possible improvements of the new proposal.

The differences between our submitted paper and the conference paper are as follows:

- In the submitted paper, we introduced the motivation and design philosophy of our protocols.
- Based on the theoretical analysis, we demonstrated two schemes for both the weak PUF and strong PUF, where the method for weak PUF is the extension of our conference paper and the method for strong PUF is a new proposal.
- The evaluation was performed on existing weak PUF and strong PUF designs to show the universality of our design compared with the DRAM-PUF-based evaluation in the conference paper.
- The runtime and successful rate of our design are further evaluated with both the weak and strong PUFs. The test results demonstrate that our proposal can reach a 100% reconstruction rate under some specific parameters with less than 1 s of locking time and a few seconds of unlocking time.
- We rigorously analyze the security of our methods using a more comprehensive lens.

## 2. Background

### 2.1. Physical Unclonable Functions

A PUF is a hardware entity that can be challenged with challenges and generate the corresponding responses [2]. Due to the random manufacturing variations, the challenge–response behavior varies between PUF instances. This unique feature can be extracted into digital form and used in a key generation, identification, and authentication scheme. Based on the number of possible CRPs, PUFs can be mainly divided into two classes: weak PUFs and strong PUFs [3].

Weak PUF is a kind of PUF that has very few or even only one CRP in one PUF instance. The most popular weak PUF design is SRAM PUFs [16]. The random power-up state of SRAM circuits existing in the embedded systems can be extracted and used as PUFs. The kind of weak PUFs that exist in off-the-shelf devices are also known as intrinsic PUFs. In addition to SRAM PUFs, a variety of other intrinsic PUFs have been proposed. DRAM is pervasively used in existing embedded systems. Prior works have shown that DRAM can be used as an attractive, low-cost intrinsic PUF in commercial off-the-shelf devices, which do not require any hardware modifications [17]. Three types of DRAM PUFs have been proposed based on: the decay feature of DRAM cells [17], startup values [18] of the DRAM cells, and the write latency [19] of the DRAM cells. To the best knowledge of the authors, in all the PUF implementations, decay-based DRAM PUFs are the only PUF implementation that is accessible at the runtime of the existing devices. Weak PUFs are considered as an attractive low-cost security anchor to mitigate some potential pitfalls in NVM-based key storage scheme. When used in the field, the CRPs should be kept in secret which is comparable to the security of a key stored in NVM.

In opposition to the weak PUF, the strong PUF can drive a more complex, even exponentially CRPs space. For the adversary, a full read-out of all CRPs or predicting the response for the new challenge based on the known CRPs in a limited time are very difficult. A well-studied example of strong PUF is the Arbiter PUF (APUF) [20]. APUF extracts the unique CRPs from the randomly distributed manufacturing variations by evaluating the runtime delays in electrical components. A *k* stage APUF consists of 2k multiplexers and one arbiter. Each stage is conditioned by a 1-bit challenge (straight or cross). When used in the field, the incoming electrical signal is split into two signals. These two signals are raced with each other in parallel along the signal transaction path determined by the challenge set $c_{1}$, …, $c_{k}$. The final response *r* (zero or one) is generated by judging which path is arrived at first by the arbiter. For the *k* stage APUF, the CRPs space is $2^{k}$. In addition to the APUF, the researchers in recent years have proposed some new strong PUF candidates, e.g., the Power Grid PUF [21], Clock PUF [22] and Crossbar PUF [23].

A typical application of strong PUFs is the CRPs-based identification and authentication [4]. The proposal is lightweight and error-tolerant. However, the freely and publicly accessible CRPs interface of that scheme made the strong PUF vulnerable to machine learning attacks [24]. Therefore, several variants of APUFs have been proposed to countermeasure the machine learning attacks. Nevertheless, even these improved architectures are not secure for some powerful modeling attacks [25]. In this paper, we demonstrate that the fuzzy exponential number of CRPs in strong PUFs can be used to protect the exponential number of secret keys in the vault which is not vulnerable to modeling attacks.

### 2.2. Helper Data Algorithm

**Figure 1:**
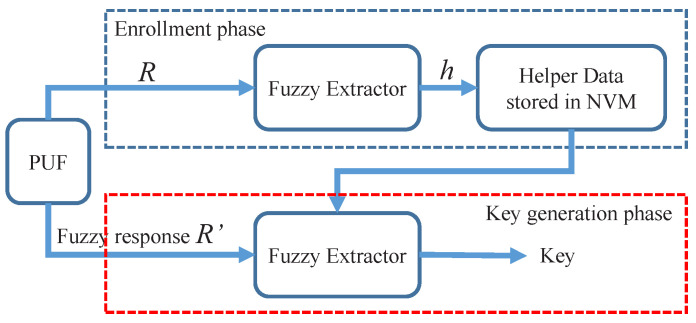
The architecture of the key generation scheme based on the fuzzy extractor algorithm.

To use PUFs as reproducible keys, a helper data algorithm, also known as Fuzzy Extractor [26], is required. As shown in Figure 1, the helper data algorithm consists of an enrollment phase and a key generation phase. In the enrollment phase, the helper data are generated by a fuzzy extractor algorithm, where *R* is the PUF response and *h* is the helper data. Helper data can be stored publicly as it should not leak any information about *R*. At the key generation phase, a fresh and fuzzy response of PUF $R^{\prime }$ is queried from the target device. Using the fuzzy extractor algorithm, the original response *R* can be regenerated by the error correction process of the helper data algorithm if the Hamming distance of *R* and $R^{\prime }$ is smaller than the error-correcting capability of the helper data algorithm. Then, the regenerated response *R* can be used to generate a reduplicated key.

There are also some improved helper data algorithms. For example, Ref. [27] proposed a two-layer helper data algorithm to avoid leakage problems due to the bias of DRAM PUF. The first layer selects the cells with higher reliability to improve noise resilience. The second layer uses pointers that identify a similar number of stable fast cells and slow cells to avoid the bias problem.

### 2.3. Fuzzy Vault Scheme

Traditional encryption is not suitable for biometric template protection due to intra-user variations and noise. An error-tolerant encryption scheme is required to encrypt the fuzzy biometric template. Fuzzy Vault is a cryptographic scheme proposed by Juels and Sudan [8] which is suitable for applications where biometric authentication and cryptography fusion are required [10]. Previous works have shown that it is an effective way for biometric template protection in biometric identification systems. In the fuzzy vault scheme, a secret key is used to protect the biometric template. In the locking process, the biometric template is locked by the secret key in an unordered real point set. The randomly selected chaff points hide the real point set from the attacker to ensure biometric template security. Although the biometric data suffer from the noise effects, the feature of error-tolerant of Fuzzy Vault allows the identification success even if part of the biometric template remains stable. The security of the vault in brute-force attacks depends on the difficulty that the attacker can tell the real points from a large number of chaff points.

One of the most popular Fuzzy Vault applications is the fingerprint-based Fuzzy Vault [10]. Fuzzy Vault can provide strong security protection by merging the fingerprint template in a lot of random noise. There are several fingerprint-based fuzzy vault implementations. Uludag et al. implemented the algorithm in MATLAB and tested the design with the public fingerprint database [28]. Ref. [29] implemented an alignment-free fuzzy vault using C++. Ref. [9] is the first fingerprint-based fuzzy vault implementation on distributed devices with comparable accuracy and efficiency. Our implementation is partly based on the implementations in paper [9].

## 3. Proposed Key Storage Method

### 3.1. Design Philosophy

**Figure 2:**
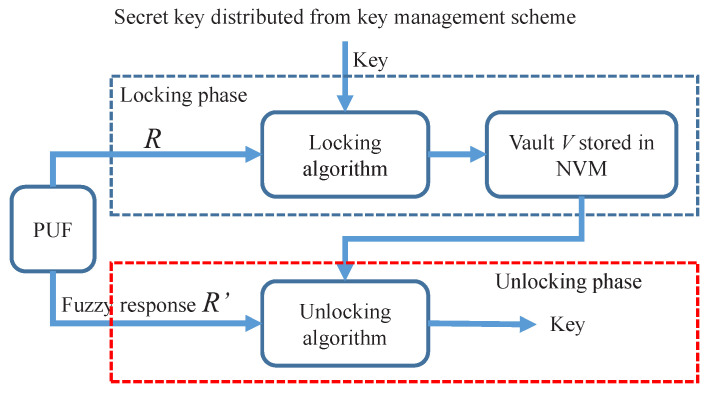
The architecture of the proposed key storage scheme.

As shown in Figure 1, the traditional key generation scheme utilizes a fuzzy extractor algorithm to remove noise (error correction) from the fuzzy responses of PUF and compress the reduplicated responses into a full entropy key (privacy amplification). The generated key can then be used to encrypt the secret data. On the contrary, as shown in Figure 2, in our proposal, the secret key was distributed from key managers or other key generation primitives. The vault is only responsible for securing the secret key with PUFs. It can be considered as an error-tolerant encryption scheme that uses PUF to encrypt the secret key.

Our basic idea is motivated by the reliability feature of the fuzzy vault scheme. The implementation of the fuzzy vault that is used to protect the biological template is based on the polynomial reconstruction strategy to countermeasure the reliability issue of the biological information. Similarly, in the locking process of our proposal, firstly, a *t* degree polynomial *P* is constructed by a secret key that needs to be protected. Then, the polynomial is evaluated by the PUF responses *R* to generate f≥t+1 real points. By these steps, the secret key is shared among *f* points. The PUF responses and the secret key are fused in this real point set. To secure these points, some chaff points can be added in the vault *V* combined with the real points which cannot be separated by the adversary. In the unlocking phase, the system tries to reconstruct the polynomial *P* (secret key) based on the freshly extracted fuzzy responses $R^{\prime }$. Due to the noise effects, there would be some differences between *R* and $R^{\prime }$. Here, we call $R^{\prime }$ as the fuzzy responses of PUF. Although noise exists in $R^{\prime }$, the reliable responses in $R^{\prime }$ are effective data to find out enough real points from *V*. Due to the error-tolerant feature of the polynomial reconstruction process, any t+1 or more points in the *f* real points that were selected by $R^{\prime }$ can recover the secret key by the Lagrange interpolation. Therefore, only part of the responses in $R^{\prime }$ are required to remain stable. This error-tolerant feature is suitable for the fuzzy responses of PUF. In this paper, we will show how weak and strong PUFs can be used to protect the secret key in the vault. It provides a new train of thought about how to protect keys in PUFs-based hardware security systems.

### 3.2. Leveraging Weak PUFs

We present our LOCK algorithm in Algorithm 1 and our UNLOCK algorithm in Algorithm 2.

Computing in GF allows mathematical operations to scramble data easily and effectively. Previous fingerprint-based Fuzzy Vaults were implemented on $2^{16}$ [10] or $2^{32}$ [9]. Many existing weak PUFs are implemented and used in 8-bit or 16-bit data width, e.g., SRAM PUF [16] and DRAM PUF [27]. Therefore, implementing our design under integer multiples of 8 or 16 is helpful for computation efficiency. However, one concern is that some weak PUFs only have hundreds of cells and each cell can generate a 1-bit binary response only. We use the Galois field $GF(2^{16})$ for constructing the weak PUF-based vault. $GF(2^{16})$ can offer a relatively sufficient large universe for the vault in security applications [30].

In the LOCK process, the secrete data *M* of length *k* in $GF(2^{16})$ are used to construct a *t* degree polynomial *P* by partitioning it into k÷16 16-bit values and considering them as coefficients of *P*. The degree of the constructed polynomial *t* should be large than 12 to offer sufficient security of the vault [31]. Therefore, if k<192(16×12), the secret data should be extended to length at east 192 by some random number. Furthermore, the data length *k* should meet the requirements of integer multiple of 16 to form $M^{\prime }$ To check the integrity of the secret data in the UNLOCK process, Cyclic Redundancy Check (CRC) [32] is chosen that encodes the extended message $M^{\prime }$ in 16-bit codewords. The CRC-16 codewords are appended to the extended message to obtain a new secret *S*. Based on the new message *S*, a *t*-th-order polynomial *P* can be constructed by partitioning *S* into t+1 parts $S_{0}\cdots S_{t}$ and considering them as the coefficients of polynomial $P\left(x\right)=S_{t}\cdot x^{t}+S_{t-1}\cdot x^{t-1}+\cdots +S_{0}$. The polynomial *P* contains all the secret messages. It will be protected by the Fuzzy PUF.

The system queries the PUFs response from the target device and obtains a measurement R=PUF(C), where *C* is the selected challenge. *C* would be the selected addresses with higher reliability cells. For example, for SRAM PUFs, in [33], the stable and uncorrelated cells in SRAM PUF were selected by the neighbor-influenced cell selection algorithm under different conditions.

Due to the algorithm operating in the finite field $GF(2^{16})$, the PUF measurement *R* is partitioned into *f* 16-bit elements $R_{1},R_{2},\cdots ,R_{f}$, where f>t+1. Treating the elements of *R* as x-coordinate values, the polynomial *P* is evaluated at all the elements of *R* to obtain a point set $Q=\{\left(R_{i},P\left(R_{i}\right)\right)|i=1,2,\cdots ,f\}$. To reconstruct the polynomial *P*, only t+1 elements in *R* need to be stable. Therefore, our proposal has the so-called error-tolerant feature, which ensures that the protected message can reproducibly be generated from the noise PUF response. As the difference between *f* and *t* becomes larger, the error-tolerant feature of our proposal will be improved. In this step, the secret data are merged into the hardware-based unique fingerprint.

The weak PUF used in our paper is a 16×16 current comparator-based PUF [34]. In the idealized case, 16 1-bit PUF cells can generate one element in *R*. Therefore, f≤16 if the cells are not reused in *R*. If the reliability of some PUF cells is relatively low, there may not be enough reliable elements in *R* that can be used to protect the secret data. One method to improve the storage capacity of vaults is to reuse some cells in the points construction process. We use a simplified method to reuse the cells and test the feasibility of our proposal. 16×16 PUF cells are numbered from 0 to 255. Group one is formed by cells from 0 to 15. Group two is moved right 1-bit and then generated by cells from 1 to 16. Therefore, the number of groups is extended from 16 to 256. It should be noted that the security of the vault is not improved with this method. Once the adversary screens out one point from the vault, he can then repeat the “1-bit move” method to brute force all the points. For weak PUFs in megabytes or even gigabytes, the process is not necessary.

The adversary may have access to the vault. Therefore, set *Q* needs to be protected to prevent attackers reconstruct the polynomial. To obscure set *Q*, some random chaff points can be added in the vault which cannot be distinguished by the attacker. The chaff points should be randomly distributed in the vault. Thus, it should be generated by a random number generator. Prior works have shown that many PUFs can be used as a true random number generator. Therefore, a natural method to generate chaff points would be to extract some random data from the intrinsic PUFs. One good choice is the DRAM PUF-based random number generator [35], which is accessible at the runtime of the commodity device without any hardware modifications. Furthermore, for the chaff points set $CF=\{\left(a_{j},b_{j}\right)|j=1,2,\cdots ,g,g\gg f\}$, the number of chaff points *g* in the vault should be much larger than the number of points (*f*) in *Q*.

Then, the resulting vault V={Q∪CF|,g≫t} is generated, where *Q* is the points with secret data that are locked by PUF and CF is the set of chaff points generated by the PUF-based random number generator. An adversary maybe has access to *V* as it is stored publicly on the device. The security of the vault *V* depends on the difficulty that the attacker can tell the real points *Q* from a large number of chaff points without any information of PUF on the device.

In the UNLOCK procedure, the device attempts to release the secret data *M* from the vault *V* by the fuzzy responses extracted from the PUF. The device queries the PUF response from the target device and obtains a fresh measurement $R^{\prime }=PUF\left(C\right)$, where *C* is the same challenge with the LOCK phase. Due to the noise effects, $R^{\prime }$ and *R* may not be equal. Like the LOCK phase, the new PUF measurement $R^{\prime }$ is partitioned into *f* 16-bit elements $R_{1}^{\prime },R_{2}^{\prime },\cdots ,R_{f}^{\prime }$. Based on the fuzzy PUF template $R^{\prime }$, a fuzzy set $Q^{\prime }=\left\{\left(R_{i}^{\prime },V\left[R_{i}^{\prime }\right]\right)|i=1,\cdots ,l\right\}$ can be selected out from vault *V* by matching $R_{1}^{\prime },R_{2}^{\prime },\cdots ,R_{f}^{\prime }$ with those of the candidate real points. The noise effects may result in the mismatching of some points. Therefore, l≠f.

Although the PUF template suffers from noise effects, the feature of error-tolerant of polynomial reconstruction allows the UNLOCK process to recover the secret data.

In order to extract the secret data, subsets of t+1 real points need to be found from the candidate set $Q^{\prime }$. As there are *l* candidate points in $Q^{\prime }$, the number of possible combination is $C_{l}^{t+1}$ In each iteration, the system selects one subset (t+1 elements) from the candidate set $Q^{\prime }$. As shown in paper [9], the random selection method is relatively faster than iterative selection and random generation. The number of iterations can be set to be the total number of possible combinations $C_{l}^{t+1}$. The polynomial $P^{\prime }$ that satisfied the CRC-16 checking is the right polynomial. The coefficients of polynomial $P^{\prime }$ are the secret data $M^{\prime }$ that the device stored in the LOCK process. The other method that can replace the CRC-16 checking is the hash algorithm. In the LOCK process, $hash(M^{\prime })$ can be stored in the NVM, and the hash value then can be used to check the right polynomial.
**Algorithm 1** LOCK process**Require:** *M*: *k*-bit message to be stored by the device; *S*: *n*-bit codewords encoded by *M*; *P*: *t*-th-order polynomial; *C*, *R*: challenge and response of PUF; *Q*: points set with *f* points; CF: points set with *g* points;1:**function**LOCK(Input: *M*)2:    Extend the secret message *M* to $M^{\prime }$3:    Encode message $M^{\prime }$ by an error detecting code(CRC-16): $S=\{M^{\prime },ENC\left(M\right)\}$;4:    $S=\{S_{0},\cdots ,S_{t}\}$;5:    Construct P(X): $P\left(x\right)=S_{t}\cdot x^{t}+S_{t-1}\cdot x^{t-1}+\cdots +S_{0}$;6:    Query PUF: R=PUF(C);7:    $R=R_{1},R_{2},\cdots ,R_{f}$;8:    Evaluate the points set $Q=\{\left(R_{i},P\left(R_{i}\right)\right)|i=1,2,\cdots ,f\}$;9:    Generate the chaff points set $CF=\{\left(a_{j},b_{j}\right)|j=1,2,\cdots ,g,g\gg f\}$;10:   Output V={Q∪CF|g≫f};11:**end function**

**Algorithm 2** UNLOCK process
**Require:** *m*: the maximum number of reconstruction processes; *V*: points set with f+g points; *M*: *k*-bit message to be stored by the device; $P^{\prime }$: *t*-th-order polynomial; *C*, $R^{\prime }$: challenge and fuzzy response of PUF; $Q^{\prime }$: points set with *l* points; CHECK: checking process of the error detection code;1:**function** UNLOCK(Input: Vailt *V*)2:    **for** j=1 to j=m−1 **do**3:          Input Vault *V*;4:          Query PUF: $R^{\prime }=PUF\left(C\right)$;5:         $R^{\prime }=\left\{R_{1}^{\prime },R_{2}^{\prime },\cdots ,R_{f}^{\prime }\right\}$;6:          Matching all the elements of $R^{\prime }$ in *V*: $Q^{\prime }=\left\{\left(R_{i}^{\prime },V\left[R_{i}^{\prime }\right]\right)|i=1,\cdots ,l\right\}$;7:          Polynomial reconstruction:8:          **for** i=0;i<lt+1;i=i+1 **do**9:             Randomly select t+1 points from $Q^{\prime }$;10:            $P^{\prime }\left(x\right)=S_{t}^{\prime }\cdot x^{t}+S_{t-1}^{\prime }\cdot x^{t-1}+\cdots +S_{0}^{\prime }$;11:            **if** $CHECK(\left\{S_{t}^{\prime },\cdots ,S_{0}^{\prime }\right\})=0$ **then**12:                Accept the polynomial;13:                $M=DEC(\left\{S_{t}^{\prime },\cdots ,S_{0}^{\prime }\right\})$;14:                Break the for loop;15:            **else**16:                Continue the for loop;17:            **end if**18:        **end for**19:        **if** any polynomial is accepted **then**20:           Break the for loop;21:        **else**22:           Continue the for loop;23:        **end if**24:    **end for**25:    Output *M* or UNLOCK failed;26:
**end function**



### 3.3. Leveraging Strong PUFs

For weak PUFs, one PUF template just can be used in one vault to protect one secret data. Otherwise, the attacker can conduct some powerful correlation attacks (see Section 4.2) to break the security vault. Therefore, there is a linear relationship between PUF size and the number of secret data that can be protected in the weak-PUF-based vault. However, for some scenario, e.g., the HTTPS internet communication based on TLS, the client needs to store at least one secret key for each web site [11,12]. Therefore, hundreds or even thousands of keys need to be protected in the client computer. A strong PUF is more suitable for this scenario.

One strong PUF can generate an exponential number of CRPs. Therefore, the selected CRPs can be used to protect an exponential number of secret data $M_{1},M_{2},\cdots ,M_{m}$ in different vaults $V_{1},V_{2},\cdots ,V_{m}$. Like the weak PUF aforementioned, the strong PUF-based storage scheme includes two main phases: the LOCK and UNLOCK process. Concerned that the strong PUF has a large number of CRPs, we extend our implementations in $GF(2^{32})$ to further improve the security of vault which has been prevented in previous works [9,10,28].

**Figure 3:**
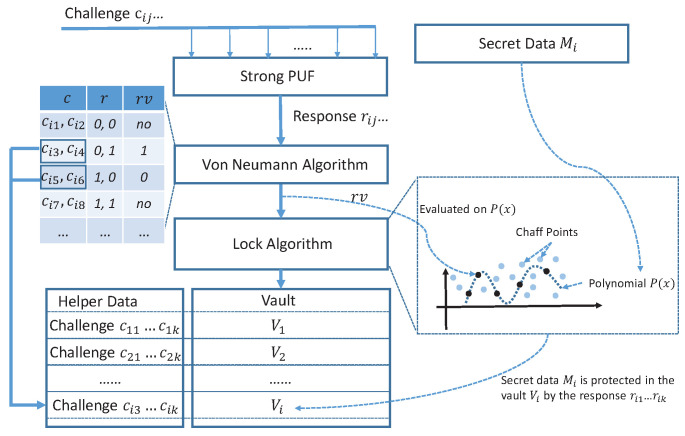
The LOCK process of strong PUF-based vault.

As shown in Figure 3, in the LOCK process, a *t*-th-order polynomial P(x) is constructed by encoding the secret message $M_{i}$ like the method shown in Section 3.2. The difference is that the coefficient of the polynomial is in the form of 32-bit, and the CRC-32 is used to encode the message. The polynomial $P_{i}$ contains all the secret messages. It will be protected by the fuzzy strong PUF.

The system queries the strong PUF by a challenge $c_{ij}$ and obtain a measurement $r_{ij}=PUF\left(c_{ij}\right)$. For some strong PUFs (i.e., APUF), $r_{ij}$ is a 1-bit response. However, the raw responses cannot be used to construct the points directly. Most of the Arbiter PUFs and the variants have a clear bias between the responses of two challenges which differ at one or more positions [36]. The bad Strict Avalanche Criterion (SAC) property would cause statistical weakness of PUFs used in our design [37]. The biased responses may cause the points degression or coincidence, which may reduce the security of the vault. Therefore, some preprocessing processes are necessary to mitigate the security issue. In TRNG design, random extractors such as XOR post-processing, parity-based von Neumann entropy extractors, LFSR-based hashing, or keyed message digest are typically required to post-process the raw response bits to obtain an unbiased random data [38,39,40,41]. As shown in Figure 3, the Von Neumann algorithm is applied to the PUF responses to produce a balanced distribution of ones and zeros. The response bit pair [0,1] is converted to an output bit 1 and the pair [1,0] is converted to 0. The response pairs [1,1] and [0,0] are discarded. Therefore, the bit rate of rv is halved or worse compared with the raw responses *r*. The new generated balanced bit stream rv is used to form the 32-bit x-coordinate value of points $R_{i}=rv_{i1},\ldots ,rv_{i16}$. The challenges that contributed to the points construction are stored as helper data (64 challenges for each point).

The polynomial $P_{i}$ is evaluated at all the elements of $R=R_{1},R_{2},\ldots ,R_{f}$ to obtain a real points set $Q_{i}=(R_{i},P\left(R_{i}\right))|i=1,2,\ldots ,f,f>=t+1$. To reconstruct the polynomial $P_{i}$, at least t+1 elements in *R* need to remain stable. Therefore, our proposal has the so-called error-tolerant feature when f>t+1, which ensures that the protected message can reproducibly be generated from the noisy strong PUF responses. In this step, the secret data are merged and protected in the hardware-based unique fingerprint. Set $Q_{i}$ needs to be protected. Some random chaff points $CF_{i}=\left\{\left(a_{j},b_{j}\right)|j=1,2,\cdots ,g,g\gg f\right\}$ can be added in the vault to improve the difficulty of brute-force polynomial reconstruction. The chaff points should be randomly distributed in the vault. One natural method to generate chaff points would be to extract some random data from a strong PUF-based random number generator [42].

Then, the resulting vault $V_{i}=\left\{Q_{i}\cup CF_{i}|,g\gg t\right\}$ is generated, where $Q_{i}$ is the points with secret data locked by strong PUF and $CF_{i}$ is the set of chaff points generated by the PUF-based random number generator. As shown in Figure 3, the vault $V_{i}$ can be stored in memory with the challenge set ci1,ci2,⋯,cik as the helper data, where k=64×f. Then, the system repeats the above process for each of the secret messages $M_{1},M_{2},\cdots ,M_{m}$.

An adversary may have access to $V_{1},V_{2},\cdots ,V_{m}$ and helper data as it is stored publicly on the device. The security of the vault depends on the difficulty that the attacker can tell the real points $Q_{1},Q_{2},\cdots ,Q_{m}$ from a large number of chaff points without any information of PUF on the device.

To improve the security of the proposal, each of the CRPs extracted from one strong PUF can only be applied once. When the secret data protected by vault $V_{n}$ are expired or promised, the system can delete the vault $V_{n}$ and all the corresponding challenges $c_{n1},c_{n2},\cdots ,c_{nk}$ in helper data. The challenges in $V_{n}$ will not be used for the remaining lifetime of the device. The uniqueness of the CRPs in each vault enables that the vault has the feature of cancellation and resisting for the correlation attacks [14].

In the UNLOCK procedure, the device attempts to release the secret data ($M_{i}$ for example) from the vault $V_{i}$ by the fuzzy strong PUF. The strong PUF needs to be measured by the corresponding challenges $c_{i1},c_{i2},\cdots ,c_{ik}$ that are stored as helper data to generate candidates subsets. As aforementioned, to reconstruct one point (32-bit x-coordinate value) requires 64 corresponding challenges in helper data. Due to the reliability issue of PUFs, the number of reconstructed bits may be less than 32. For example, in the LOCK process, the raw responses of strong PUF are 0,1 with challenges $c_{2},c_{3}$. By the Von Neumann algorithm, the output is 1. In the UNLOCK field, due to the noise, raw responses may be changed from [0,1] to [1,0], [0,0] or [1,1]. If [0,1]→[1,1] or [0,1]→[0,0] occurs, for the Von Neumann algorithm, the bit pairs 0,0 and 1,1 will be discarded and there will be no output data. Then, the length of the candidate x-coordinate value will be less than 32. The error can be detected by the system and some compensation methods can be used. One simplified method is to consider 0,0 as 0 and 1,1 as 1. In this way, the error can be corrected in 50%. The other useful method is to re-query the strong PUF by $c_{2},c_{3}$. In our design, the majority voting method is used to regenerate rv with higher reliability when the Von Neumann algorithm detects any errors. The process can be seen as a kind of error detection for strong PUFs, which may be useful for other authentication scenarios. The secret data then can be regenerated when at least t+1 out of *f* points remain stable by the method used for weak PUF.

## 4. Security Analysis

In our proposal, the adversary has access to the vault and helper data as it is stored publicly on the device. As a secret data storage scheme, the CRPs interface of both weak and strong PUFs in our proposal needs to be access-restricted, even if the adversaries have PUF-carrying devices. For weak PUFs, access restriction is a basic security requirement in nearly all the application scenarios [3]. On the contrary, for strong PUFs, most of the application scenarios assumed that the device has an unprotected CRPs interface. In our proposal, the strong PUF can be considered as a hardware entity that has an exponential number of “weak PUFs”. Each “weak PUF” with few and fixed challenges is used to protect one secret datum in one vault. The vault will be compromised if the adversaries can challenge the “weak PUF” with the challenges in the helper data which is stored publicly. For some strong PUF instances, the collected CRPS by the physical access can be used to model the strong PUF behavior which can then be used to predict the new responses for the known challenges. Therefore, both the existing vaults and the vaults that will be generated in the future can be compromised. To countermeasure this issue, the erasable PUF technique can be used to block the physical access of strong PUFs on the hardware level, which is a generic method for any given silicon strong PUF with a digital CRP-interface [43].

### 4.1. Brute Force Attack

The security of the vault relies mainly on the number of chaff points and real points. It can be evaluated using Equation (1), where *f* is the number of real points formed in the LOCK phase, *t* is the degree of the polynomial, and *g* is the number of chaff points. $H_{\infty }$ roughly corresponds to the same level of difficulty in brute-force attacking a full-entropy password.

(1) $$H_{\infty }=\left(Q|V\right)=-log\left(\frac{C_{f}^{t+1}}{C_{g+f}^{t+1}}\right)$$

For a message *M*, the security of the vault can be improved by constructing a polynomial with a higher degree *t* in the LOCK process. The method is to append more random bits on *M*. However, higher *t* means more runtime both in the LOCK and UNOCK process and a lower unlock successful rate (shown in Section 6.2). Formula (1) also shows that the entropy of the vault improves as the number of chaff points *g* increases because the adversary must guess more times in the brute-force attack ($g↑\to H_{\infty }↑$). However, more chaff points mean that the system needs more time for chaff point generation (shown in Section 6.2) and more memory resources for vault storage. Furthermore, although the increasing real points can improve the UNLOCK successful rate, the security of the vault decreased because the adversary has higher possibilities to select enough real points from the vault ($f↑\to H_{\infty }↓$). More real points require more memory space to store the vault and helper data. Therefore, the vault is a tradeoff between security, reliability, runtime, and resource consumption.

Additionally, the points in the vault should be randomly distributed. The aggregately distributed points will reduce the security of the vault. The probability of successfully unlocking the secret data will increase when the adversary randomly brute forces some specific area of the vault. Here, we show an extreme case: The vault is divided into *n* parts. Each part has $\frac{f+g}{n}$ points. We assume that all the real points are distributed in one of the *n* parts. The number of combinations of brute-force attack becomes $n\times C_{\frac{g+f}{n}}^{t+1}$ from $C_{g+f}^{t+1}$. Compared with Equation (2), the security of the vault should drop to:

(2) $$H_{\infty }^{\prime }=\left(Q|V\right)=-log\left(\frac{C_{f}^{t+1}}{n\times C_{\frac{g+f}{n}}^{t+1}}\right)$$

If g+f=100, t+1=7, f=10, and n=2, the security of the vault decreases from $H_{\infty }=27$ to $H_{\infty }^{\prime }=20$. Therefore, the real points should be distributed randomly in the vault to maintain the security value in theory. For a weak-PUF-based vault, the randomness feature of weak PUF is required to mitigate the possible bias, which is comparable to the PUF-based key generation scheme. For strong PUFs, the systematic bias of different delay stages needs to be concerned, which is common for the raw response bits generated by most delay-based PUFs. The Von Neumann algorithm used in our proposal (as shown in Section 3.3) can reduce the points aggregation or overlap introduced by PUFs bias.

Furthermore, besides the PUF bias, an unsuitable challenge generation method may further introduce the points aggregation or even coinciding. For example, point $po_{1}$ is extracted by *n* challenges $C_{11},C_{12},\cdots ,C_{1n}$ and point $po_{2}$ is generated by the challenge set $C_{21},C_{22},\cdots ,C_{2n}$. Each challenge $c_{ij}$ is a 64-bit set $c_{ij}=\left\{c_{ij}\left[0\right],c_{ij}\left[1\right],\cdots ,c_{ij}\left[63\right]\right\}$. The strong PUF has a systematic bias of different delay stages (e.g., bad SAC property), which is common for the raw response bits generated by most delay-based PUFs. The stage that is closer to the arbiter, e.g., $c_{ij}\left[63\right]$ has a relatively stronger influence on the PUF’s output. Some bit flips on the stages that are far from the arbiter may have very little or even no influence on the PUF’s output. For $po_{1}$ and $po_{2}$, one extreme case is that the differences between $C_{11},C_{12},\cdots ,C_{1n}$ and $C_{21},C_{22},\cdots ,C_{2n}$ all come from the stages that are very far from the arbiter, e.g., c[0] and c[1]. For example, the challenges are generated by accumulation algorithms. The points constructed by these responses would degrade or even coincide with a certain degree of probability. Some preprocessing or detection module is necessary for that strong PUF to deal with the problem. In our proposal, we leverage a random number generator to generate randomly distributed challenges to mitigate the problem to some extent.

### 4.2. Correlation Attack

A Correlation Attack [44] is an attack in which the adversary can obtain at least two vaults that belong to the same PUF template. Although these vaults are created by different secret data and different chaff points, the X-ray values of the real points should highly overlap since they are generated by the same PUF responses [14]. Therefore, some chaff points will be filtered out and the attacker could obtain the real points easier.

Therefore, one weak PUF instance only can be used to protect a few or even only one secret datum because each weak PUF has very few or even only one CRP. For the new data that need to be protected, the system needs to find or implement more weak PUF instances. Paper [10] demonstrates an unlinkable Fuzzy Vault for multiple fingerprints, providing an acceptable security level against offline attacks. This method is also suitable for our proposal. There are several components in one embedded system that can be used as weak PUF to protect the secret data, e.g., SRAM PUF, DRAM PUF, and Nandflash PUF. Ref. [45] also proposed that embedding some fake polynomials in the vault can increase the security of secret polynomials further. In addition, some improved Fuzzy Vault scheme [46,47] can be used to validate the scheme.

For a strong PUF instance, one CRP can be used to protect secret data only once. The exponential number of CRPs can help one strong PUF to protect an exponential number of secret data. For a 64-bit strong PUF used in our paper, there are $2^{64}$ CRPs that can be used in theory. For example, 64×f CRPs (considering the Von Neumann algorithm) are required to form *f* real points in one vault. $\frac{2^{64}}{64\cdot f}$ vaults can be generated to protect $\frac{2^{64}}{64\cdot f}$ secret data. Therefore, strong-PUF-based vaults are not vulnerable to correlation attacks.

### 4.3. Modeling Attack for Strong PUFs

For some strong PUF instances, the collected CRPs can be used to model the strong PUF behavior which can then be used to predict the new responses for the known challenges. Therefore, if the strong PUF is modeled, both the existing vaults and the vaults that will be generated in the future can be compromised.

The basic premise of a modeling attack is that an adversary can collect a large number of CRPs of a given strong PUF. However, the application scenario of our proposal is similar to the weak PUF-based key generation scheme. The modeling attacks on strong PUFs are not applicable to the strong PUFs in our proposal. At first, in our scheme, the application of strong PUF is to derive CRPs inside the hardware system. The CRPs are not exposed to the unsecured channel between devices and servers. The adversary cannot capture the CRPs outside the system such as the CRPs-based authentication scheme [2]. Second, inside the system, in addition to the security channel, the response interface of strong PUF can be access-restricted both for the hardware and software. The LOCK and UNLOCK processes should be conducted in the security hardware or software boundary, e.g., the Trusted Platform Module (TPM) or Secure Enclave Processor, to prevent possible attacks from malicious code. Furthermore, invasive attacks should be prohibited. Although the responses exist in the system only for a short time, if the responses are read out by some invasive attacks, the security scheme will be compromised. Therefore, the strong PUF used in our scheme is not vulnerable to the modeling attack if the system can provide some basic security requirements which are in principle comparable to the security of the existing security systems.

## 5. Experimental Setup

Our proposal is not designed for any specific PUFs. There is a tradeoff between reliability, security, and runtime in our design. For example, the error-tolerant feature of our proposal can compensate for the reliability issue of PUFs to some extent. As the difference between the number of real points (*f*) and polynomial degree (*t*) becomes larger, the error-tolerant feature of our proposal will be improved. However, more real points need more resources and runtime and can reduce the security of the vault to some extent. Therefore, discussions about these features based on some PUF examples are necessary. This section shows some basic features of our proposal evaluated on two existing PUF designs: current-comparator-based weak PUF [34] and current-starved-inverter-based strong PUF [48] implementations.

The weak PUF used in our paper is a 16×16 current comparator-based PUF [34]. The manufacturing variations of one customized current comparator are extracted to generate a 1-bit response. In the PUF design, the reliability is improved by introducing the proportional to absolute temperature (PTAT) characteristic together with the positive feedback mechanisms. Furthermore, the design has relatively better energy efficiency compared with some existing related works. Our design is validated by the prototype chips fabricated in a standard 65 nm CMOS process in different environments. Paper [34] has shown that the average reliability of the current comparator PUF is 99.05% and the 256-bit response can pass the NIST randomness tests. Here, we will study the feasibility of the PUF design used in our proposal.

The strong PUF design that we used is a 64-bit energy-efficient implementation of APUF with enhanced temperature stability by inserting current-starved inverters and using a symmetric RS-latches-based arbiter [48]. Our test is conducted based on the responses measured from the prototype chips fabricated in a standard 65 nm CMOS process.

**Figure 4:**
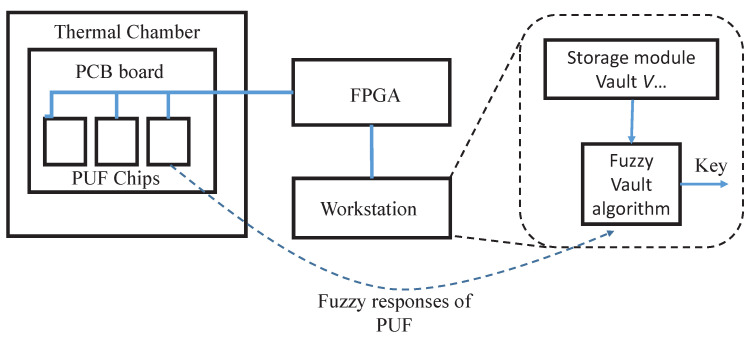
Experimental Setup.

Figure 4 presents a schematic of the experimental setup used to test the reliability of our proposal. The setup includes the following equipment: a thermal chamber (EspecSU262) which can provide stable and controllable temperature from 40 to 150 °C; a DC power supply (KeysightE3631A) that is used to provide stable power for the PUF chips; and an Altera DE2 FPGA board that communicates with the PUF chip to provide challenges and capture the responses. Inside the thermal chamber, ten weak PUF chips and ten strong PUF chips are placed for experiments under controlled temperature and voltage. The FPGA boards can communicate with the workstation via serial ports. The workstation further runs Python scripts for LOCK and UNLOCK processes.

In the python scripts, the “sympy Galoistools” is included in our implementations for polynomial evaluation and we operate the Lagrange interpolation in the Galois field. In the LOCK process, the secret data and chaff points are all randomly generated by the python scripts. The generated vault is stored in a .txt file. In the UNLOCK process, the vault is read out from a .txt file and unlocked by the new PUF query. The following parameters in our implementations can be configured to achieve different security and error-tolerant feature: polynomial degree *t*, the number of real points *f*, and the number of chaff point *g*.

## 6. Experimental Results and Discussion

The reliability of our proposal depends on the robustness of the PUF template and the error-tolerant feature of the vault. Under a certain PUF template’s reliability, the error-tolerant parameters can be selected to meet the reliability requirements of the vault. To understand the reliability of our proposal, we evaluated the selected weak and strong PUFs’ reliability using the thermal chamber.

### 6.1. The Test Results for Our Proposal Based on Weak PUF

The weak PUF used in our paper is a 16×16 current-comparator-based PUF. As aforementioned in Section 3.2, 16 PUF cells can generate one point. Even if one cell in the points is unreliable, the points will be seen as unstable points. In our tests, the reliability tests are in the form of a cells group. Each group has 16 cells, which represent one point.

**Figure 5:**
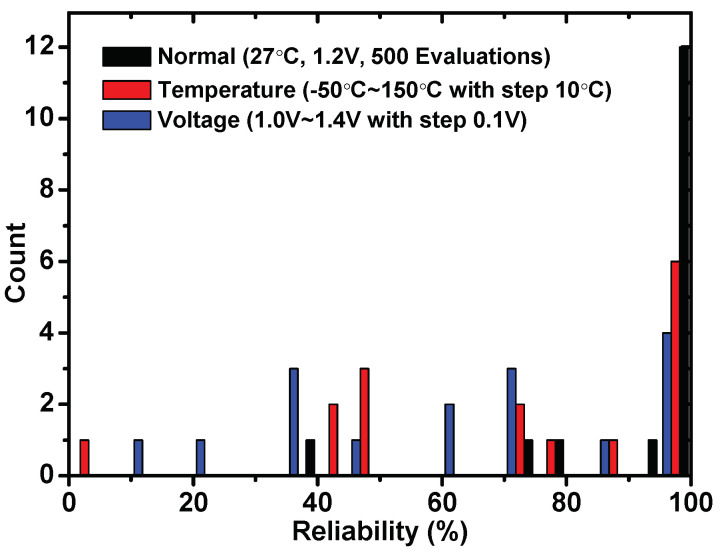
The reliability of weak PUFs in 16-bit groups.

For a weak PUF with 16×16 cells, it can generate 16 groups naturally, and the groups are numbered from 1 to 16. As demonstrated in Figure 5, the reliability for each group is calculated from more than 4000 tests with the operating temperature in room temperature, temperature ranging from 40 to 150 °C and the supply voltage ranging from 1.0 V to 1.4 V. Here we choose the response at 1.2 V, 27 °C as the reference. Each bar in the figure represents the number of groups under a certain reliability.

This feature ensures that the security of the vaults can be evaluated by Formula (1). Therefore, the parameters of the vaults can be set according to the working environment. For the system operating under room temperature, we have 12 points with 100% reliability. These points can be selected as the real points to protect the secret data. The other points also can be used as real points. However, we need to make sure that there are enough real points that remain stable in one UNLOCK process. If the system operated under extreme temperature conditions which vary from −40 °C to 150 °C, only seven points remain stable in all the tests. If the polynomial degree in the vault is larger than 7, e.g., the secret data are divided into 14 parts and generate a 13-stage polynomial, the proposal would be not useful due to insufficient reliable points. Similarly, in voltage unstable conditions, only four points remain stable. Furthermore, the Hamming distance between all the points extracted from 10 chips is tested. There are no overlapping points (no equal points).

**Figure 6:**
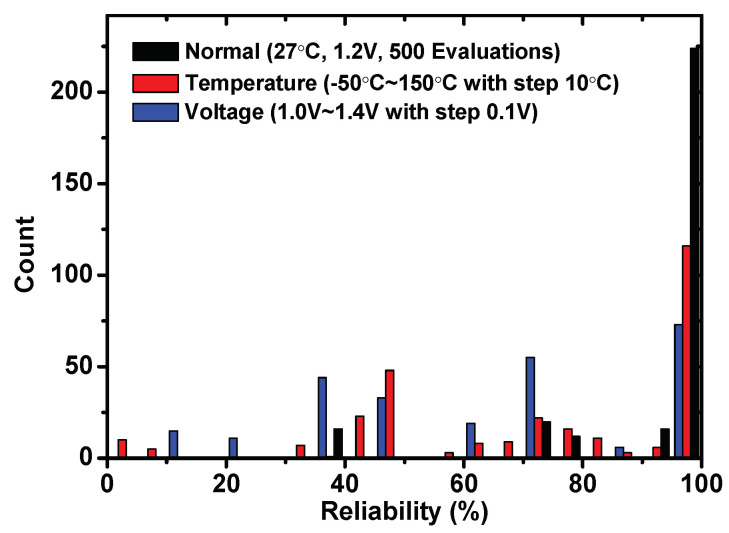
The reliability of weak PUFs in 16-bit groups with reused cells.

Actually, as shown in paper [34], most of the cells remain stable even in some extreme test conditions. The small number of unreliable cells caused most of the real points to be unstable. One natural method to improve the system’s reliability is to filter out unreliable cells and use the reliable cells to construct real points. Figure 6 shows the reliability of 256 groups under different test conditions when the cells can be reused. Even under the worst case (voltage changes from 1.0 to 1.4 V), the system still has 50 reliable groups. Furthermore, the Hamming distance between all the points extracted from 10 chips is tested. Although the cells are reused in some points, there are no overlapping points, which benefits from the randomness of weak PUF used in our paper. Therefore, more secret data can be protected in one vault.

Table 1 indicates that the UNLOCK successful rate varies significantly for different parameters. In the LOCK process, we set the polynomial degree t=12. In the table, the unlock successful rate with 15 and 20 real points is tested by randomly selecting 15 and 20 16-bit cell groups from the 256 groups aforementioned. For the column with 20 selected real points, 20 16-bit cell groups are selected from the groups with 100% reliability in Figure 6. To further improve the UNLOCK successful rate, the majority voting method is used with different query times. The impact of each parameter combination is analyzed by operating 1000 UNLOCK processes each. The test results indicate that a higher number of real points and a higher number of majority voting times can help to improve the success rate. The cells with higher reliability that filtered out in the LOCK process also increase the vault reliability.

### 6.2. The Test Results for Our Proposal Based on Strong PUF

The strong PUF used in our paper is a 64-bit current current-starved-inverter-based APUF. As aforementioned in Section 3.3, 64 1-bit PUF responses can be used to generate one point. Even if one response in the points is unreliable, the points will be seen as unstable points. In our tests, the reliability tests are in the form of a CRPs group. Each group has 64 randomly selected CRPs which represent one point.

**Figure 7:**
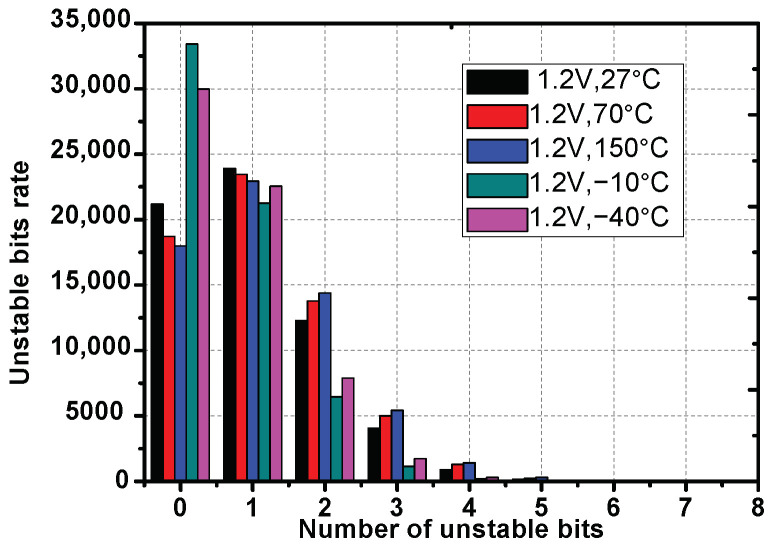
The reliability of APUFs in 32-bit response groups.

In the experimental setup, we query the strong PUF under different temperatures: −40 °C, −10 °C, 27 °C, 70 °C, 150 °C for 10 chips. For each test condition, we do 100 times PUFs query for $10^{6}$ random challenges. The corresponding responses can generate 62,500 CRPs groups. Figure 7 shows the reliability under different test conditions referred to as the tests under 27 °C. The horizontal axis in the figure represents the number of unstable CRPs in one group, and the vertical axis represents the number of groups. The worst case is the tests under 150 °C. About 17,500 in 62,500 groups remain stable. When used in the field, to protect the secret data in a *t*-stage polynomial in a vault with *f* real points, at least t+1 points should be selected from the 17,500 reliable groups. The rest of the f−t−1 real points can be selected from the other groups. The secret data can then be unlocked when at least t+1 points in *f* remain stable. Furthermore, the Hamming distances between all the points extracted from 10 chips are tested. There are no overlapping points, which benefits from the randomness of the strong PUFs used in our paper.

It should be noted that although some points can be pre-selected by extreme tests, the system cannot make sure that the so-called reliable points remain stable for the whole lifetime of the device. Some factors, e.g., aging, may affect the reliability of some points from reliable to unreliable or from unreliable to reliable. Furthermore, in our tests, some so-called unreliable points only have very few or even one unreliable response in 100 tests under some extreme conditions. These points also can remain stable with very high probabilities. Therefore, the parameter *f* and the method to generate real points should be adjusted based on the PUFs feature and the working conditions. In our design, a majority voting technique was used to further improve the reliability of the vault.

**Figure 8:**
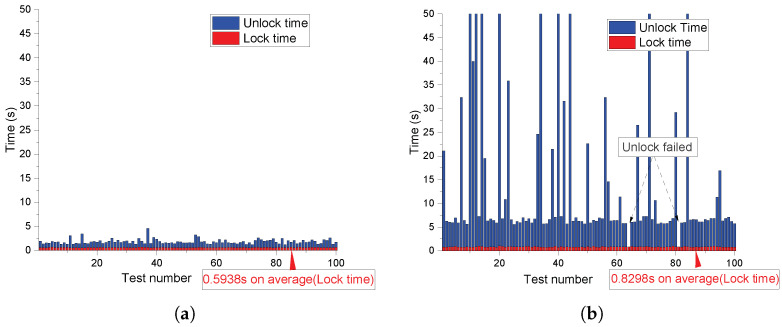
Impact of polynomial degree on unlocking rate and runtime (in seconds). (**a**) Unlocking rate and runtime test under t=12,f=40,g=300; (**b**) Unlocking rate and runtime test under t=30,f=40,g=300.

**Figure 9:**
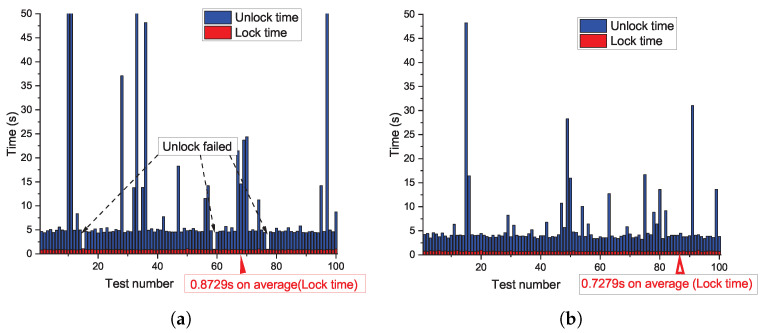
Impact of real points on unlocking rate and runtime (in seconds). (**a**) Unlocking rate and runtime test under t=20,f=25,g=300; (**b**) Unlocking rate and runtime test under t=20,f=45,g=300.

**Figure 10:**
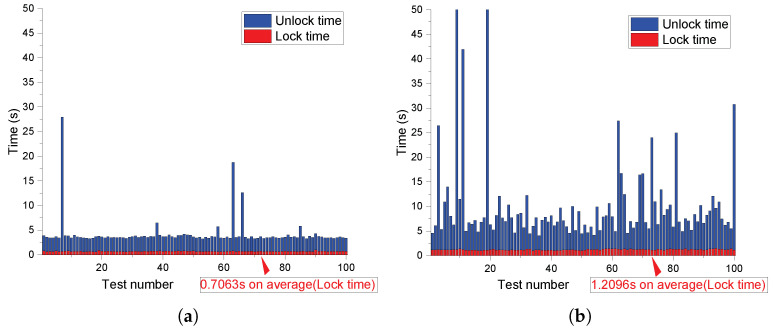
Impact of chaff points on unlocking rate and runtime (in seconds). (**a**) Unlocking rate and runtime test under t=20,f=40,g=100; (**b**) Unlocking rate and runtime test under t=20,f=40,g=500.

To evaluate the UNLOCK successful rate and runtime performance of our design, we operate the whole LOCK and UNLOCK process based on the CRPs queried from strong PUF on a PC instance with Intel i7-6700HQ CPU at 2.6 GHZ. Python3 is used to compile our Python scripts. As shown in Figure 8, Figure 9 and Figure 10, the polynomial degree, number of real points, and number of chaff points are included as control variables, respectively, in each figure. We conducted 100 times LOCK and UNLOCK processes under each parameter combination, which is shown by the abscissa in each sub-figure. The bar indicates the runtime of LOCK and UNLOCK processes in red and blue, respectively. The figures show that the UNLOCK successful rate and runtime vary for different configurations. The test results demonstrate that our proposal can reach a 100% unlocking rate by parameter adjustment with less than 1 s of locking time and a few seconds of unlocking time.

### 6.3. Discussion and Comparisons

Table 2 compares our methods against several related PUF-based key generation and storage schemes. The key generator methods proposed in [49,50,51] are all constructed by the error correction feature of the fuzzy extractor algorithm. Some other error correction methods have also been used to address the reliability issue of the fuzzy PUF response [52,53,54,55]. Most of the existing works are focused on the key generation scenario which is different from our design. Only paper [56] and our work tried to protect the key through the fuzzy PUF response. Ref. [56] uses the fuzzy extractor algorithm to generate a key. Then, the generated key is used to encrypt the received key that needs to be protected. Compared with the key generation scheme that is based on a fuzzy extractor, our proposal needs more time to unlock the key. One reason is that our proposal needs many more rounds to select enough real points based on the fuzzy responses of PUF. From the security analysis and experimental results, we can see that there is a tradeoff between security, unlocking time, and reliability. The parameters can be selected in different application scenarios. Furthermore, the efficiency of our methods can be further improved by the hardware accelerator.

The higher polynomial degree *t* requires more runtime to evaluate each real point in both the LOCK and UNLOCK process, although the security can be improved (as shown in Section 4). In each UNLOCK iteration, t+1 real point candidates are randomly selected from the vault to unlock the secret data until the unlocked secret data meet the CRC-16 checking. A higher *t* indicates that more reliable real points need to be selected in each iteration. Therefore, the iteration is more likely to fail the CRC-16 checking because of the strong PUF noise. More iterations led to more prominent changes from Figure 8, Figure 9 and Figure 10 or even failed the UNLOCK process.The higher number of real points *f* indicates that the system needs more time for more polynomial evaluation both in the LOCK and UNLOCK processes. In the UNLOCK process, the reliability requirements for PUFs is at least t+1 points; the real points candidates remain stable. For a certain t+1, more real points candidates reduce the requirements for PUF’s reliability ($f↑\to \frac{t+1}{f}↓$). Therefore, the successful rate increases from Figure 8, Figure 9 and Figure 10. For the chaff points, it is more about the security issue as talked about in Section 4. The runtime of the LOCK process increased due to the increasing chaff points. In the UNLOCK process, more chaff points mean a higher probability that more chaff points can be selected as real points candidates unexpectedly due to the noise of PUF responses.

## 7. Conclusions

In this work, we presented that the short secret data can be protected by the fuzzy PUF template with an error-tolerant encryption algorithm. We implemented our proposal in Pythons and evaluated the runtime and unlock successful rate based on existing weak and strong PUF designs. The evaluation of our proposal showed its feasibility. We further presented the security threats of our proposal as well as the possible solutions based on the feature of PUFs. This paper gives a new train of thought about PUFs-based data protection schemes.

## Figures and Tables

**Table 1:** Unlocking rate of weak PUFs under different parameters.

Majority Vote	Number of Real Points		
	15	20	20 with Selected Cells
1	32.69%	76.68%	97.98%
4	50.70%	88.34%	97.09%
8	58.88%	93.31%	100%
16	64.65%	94.88%	100%
32	65.68%	95.56%	100%
64	77.77%	98.38%	100%
128	0.85553%	98.75%	100%

**Table 2:** Comparison of PUF-based key generation and storage schemes.

Works	Error Correction	Applications	PUF Types	Time
PUFKY [49]	fuzzy extractor	Key generation	weak PUF	5.62 ms on FPGA
key generator [50]	reverse fuzzy extractor	key generation	weak PUF	∼38.5 ms on software
key generator [52]	pattern matching	key generation	strong PUF	-
key generator [51]	soft-decision fuzzy extractor	key generator	weak PUF	-
key generator [53]	built-in self-test	key generator	strong PUF	1.15 us on ASIC
key generator [54]	per-device configuration	key generator	weak PUF	-
key generator [55]	polar codes	key generator	weak PUF	∼
key storage [56]	fuzzy extractor	key storage	weak PUF	∼4 s on software
our work	fuzzy vault	key storage	weak and strong PUF	∼1 s on software

## Data Availability

Not applicable.
